# Cesium Doping for Performance Improvement of Lead(II)-acetate-Based Perovskite Solar Cells

**DOI:** 10.3390/ma14020363

**Published:** 2021-01-13

**Authors:** Min-Seok Han, Zhihai Liu, Xuewen Liu, Jinho Yoon, Eun-Cheol Lee

**Affiliations:** 1 Department of Nano Science and Technology, Graduate School, Gachon University, Gyeonggi 13120, Korea; hanminsuk7@naver.com (M.-S.H.); arelaII5960@gmail.com (X.L.); wlsgh9838@naver.com (J.Y.); 2 School of Opto-Electronic Information Science and Technology, Yantai University, Yantai 264005, China; zhliu@ytu.edu.cn; 3 Department of Physics, Gachon University, Gyeonggi 13120, Korea

**Keywords:** perovskite solar cells, performance improvement, lead acetate, cesium doping

## Abstract

Lead(II)-acetate (Pb(Ac)_2_) is a promising lead source for the preparation of organolead trihalide perovskite materials, which avoids the use of inconvenient anti-solvent treatment. In this study, we investigated the effect of cesium doping on the performance of Pb(Ac)_2_-based perovskite solar cells (PSCs). We demonstrate that the quality of the CH_3_NH_3_PbI_3_ perovskite film was improved with increased crystallinity and reduced pinholes by doping the perovskite with 5 mol% cesium. As a result, the power conversion efficiency (PCE) of the PSCs was improved from 14.1% to 15.57% (on average), which was mainly induced by the significant enhancements in short-circuit current density and fill factor. A PCE of 18.02% was achieved for the champion device of cesium-doped Pb(Ac)_2_-based PSCs with negligible hysteresis and a stable output. Our results indicate that cesium doping is an effective approach for improving the performance of Pb(Ac)_2_-based PSCs.

## 1. Introduction

Organometallic halide perovskites (APbX_3_, in which A = methylammonium (MA^+^) or formamidinium (FA^+^), and X = Cl^−^, Br^−^, or I^−^) have attracted considerable attention because of their tunable bandgap, high light absorption coefficient, and long exciton diffusion length over one micrometer [1]. Since being first reported by Kojima et al. in 2009 [2], perovskite solar cells (PSCs) have been intensively investigated, with rapid improvement in power conversion efficiency (PCE) to above 25.5% [3,4,5,6,7,8]. As a result, PSCs are considered one of the most promising candidates for next-generation solar energy devices.

To prepare a CH_3_NH_3_PbI_3_ perovskite, typically lead iodide (PbI_2_) is used as the lead source, which chemically reacts with methylammonium iodide (MAI) at a molar ratio of 1:1 [9]. However, in order to obtain a high-quality perovskite film with a uniform morphology, an anti-solvent treatment is needed for the one-step spin-coating process [10]. This anti-solvent treatment requires expensive technology for the stable crystallization of the perovskite grains, which is detrimental to commercialization [11]. To overcome this problem, lead chloride (PbCl_2_) can be employed to replace PbI_2_ as the lead source, which has resulted in PSCs with similar performance to those prepared from PbI_2_ [12]. However, to fully remove the residual MACl from the perovskite film, a lengthy thermal annealing process (of about two hours) is required, which consumes a large amount of energy [13]. Besides lead halides, lead acetate (Pb(Ac)_2_) is another important lead source, which can avoid the need for the inconvenient anti-solvent treatment and lengthy thermal annealing processes [13]. Zhang and coworkers have shown that the Pb(Ac)_2_-processed CH_3_NH_3_PbI_3_ perovskite shows a more uniform and compact morphology with increased crystallinity, compared with PbCl_2_- or PbI_2_-processed perovskites. As a result, the PSCs based on Pb(Ac)_2_-processed perovskite films were shown to exhibit a high PCE of 14.7%, which is higher than that of either PbCl_2_- or PbI_2_-based PSCs [13]. To improve the performance of PSCs, morphology control of the perovskite film is crucial, because it is strongly related to the charge generation and dissociation properties of the PSCs [14]. Solvent engineering is a widely used technique for controlling the morphology of perovskite films. For example, the use of additional dimethyl-sulfoxide has resulted in an improved film morphology of Pb(Ac)_2_-based perovskite [15]. Doping of the perovskite crystal is another effective way to improve the morphology of the perovskite films. For example, Br^−^ and FA^+^ have been used to partially replace I^−^ and MA^+^ in the perovskite structure for preparing mix-cation perovskite (FA_x_MA_1-x_PbI_y_Br_3−y_), which resulted in a significantly improved PCE and enhanced stability of the PSCs [16]. For PbI_2_-processed perovskite, cesium doping into the MA site has been demonstrated to be an efficient way to improve the performance of the associated PSCs [16]. M. Saliba and coworkers have demonstrated that doping with 5 mol% cesium resulted in a uniform and compact perovskite film morphology with fewer pinholes, which significantly improved the PCE of the PSCs from 16.37 to 19.20% [16]. However, there has not been any investigation into the cesium doping effect on the film morphology of Pb(Ac)_2_-based perovskite and the performance of Pb(Ac)_2_-based PSCs. Considering the importance and convenience of using Pb(Ac)_2_ for perovskite preparation, it is crucial to dope Pb(Ac)_2_-based perovskite with cesium for PSC fabrication.

In this work, we doped the Pb(Ac)_2_-based perovskite with cesium by adding a small amount of cesium iodide (CsI) into the perovskite precursor. After doping, the perovskite film showed a uniform morphology with enhanced crystallinity and reduced pinholes, which is beneficial for charge transportation. Consequently, the champion device PCE raised from 15.22 to 18.02% with negligible hysteresis and a stable output which is a significant improvement in open-circuit voltage (V_oc_), short-circuit current density (J_sc_), and fill factor (FF) via cesium doping. Additionally, the average PCE of the Pb(Ac)_2_-based PSCs was significantly improved from 14.1 to 15.57%. Our results demonstrate the superior effect of cesium doping on the performance improvement of Pb(Ac)_2_-based PSCs.

## 2. Materials and Methods

### 2.1. Materials

Poly(3,4-ethylenedioxythiophene) polystyrene sulfonate (PEDOT:PSS, P AI 4083) was bought from Heraeus Co. (Hanau, Germany). 6,6-phenyl C61-butyric acid methyl ester (PCBM) was purchased from Nano-C Inc. (Westwood, MA, USA). 2,9-dimethyl-4,7-diphenyl-1,10-phenanthroline (BCP) was obtained from Xi’an Polymer Light Technology Corp (Xi’an, China). CsI was purchased from Sigma Aldrich (St. Louis, MO, USA). Methylammonium Iodide (MAI) was provided by Great Cell Solar (Queanbeyan, Australia). Pb(Ac)_2_ was provided by Tokyo Chemical Industry (Tokyo, Japan).

### 2.2. Device Fabrication

Firstly, we cleaned patterned indium tin oxide (ITO)-glass substrates sequentially in detergent, acetone, and 2-propanol for 15 min. The hole transport material, PEDOT:PSS, was deposited on the ITO-glass substrates through a spin-coating process and then annealed at 140 ℃ for 15 min in air. The perovskite precursors were made by mixing Pb(Ac)_2_ and MAI in 1 M dimethylformamide. Then, the perovskite precursor with *x* mol% cesium, where *x* = 0, 2.5, 5.0, 7.5, or 10.0, was spin-coated on the ITO/PEDOT:PSS substrates at a rotation speed of 4000 rpm, which was followed by annealing at 80 °C for 10 min. The electron transport layer (ETL) was formed by spin-coating of PC_61_BM (30 mg·ml^−1^ in Chlorobenzene) on the perovskite layer at 2000 rpm for 30 s. For better charge transport, we deposited a BCP layer onto the ETL by spin-coating BCP solution (0.5 mg·ml^−1^ in 2-propanol) at 450 rpm for 30 s. Finally, the deposition of an 80 nm silver electrode was achieved with thermal evaporation under a high vacuum of approximately 10^−6^ Torr. The device area was determined by the overlapped rectangle between the ITO and Ag electrode bars, being 0.06 cm^2^ (0.2 cm × 0.3 cm).

### 2.3. Measurements

X-ray diffraction (XRD) and X-ray photoelectron spectroscopy (XPS) were conducted for the perovskite samples using the D8 Advance X-ray diffractometer (Bruker, Billerica, MA, USA) and the K-Alpha X-ray photoelectron spectrometer (Thermo Electron, Waltham, MA, USA), respectively.

Current density–voltage (J–V) curves of the PSCs were obtained using the 2400 Series *J*–*V* Source Meter (Keithley Instrument, Solon, OH, USA) under an irradiation intensity of 100 mW cm^2^ (AM1.5). We used a solar simulator (XES-301S, SAN-EI ELECTRIC, Osaka, Japan) for simulating sunlight irradiation.

The space charge limited current (SCLC) of a hole-only device (glass/ITO/PTAA/Perovskite/PTAA/Ag) was obtained using the Keithley 2400 Source Meter under dark conditions. Electrochemical impedance spectroscopy (EIS) of the PSCs was performed with an electrochemical work station (CH instruments, Austin, TX, USA) under dark conditions. Steady-state photoluminescence (PL) spectroscopy was conducted using FLS920 (Edinburgh Instruments, Livingston, UK) at wavelengths between 720 nm and 800 nm with the excitation wavelength of 514 nm. Ultraviolet–visible absorption spectroscopy was performed with a UV–vis-NIR 3600 spectrometer (Shimadzu, Kyoto, Japan). The morphology of the devices was measured by the scanning electron microscope (SEM, JOEL, Tokyo, Japan) and atomic force microscope (AFM, Veeco, Plainview, NY, USA).

## 3. Results

**Figure 1:**
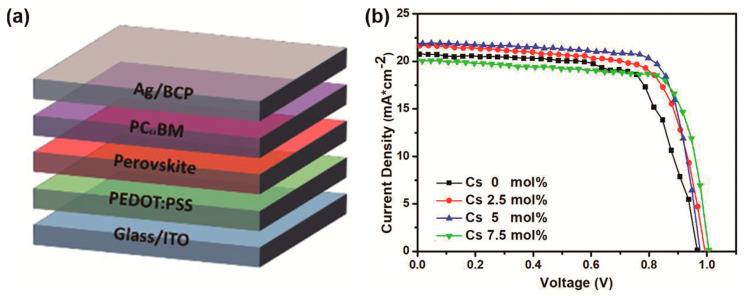
(**a**) Perovskite solar cell structure. (**b**) Current density–voltage (J–V) characteristics of the MAPbI3 PSCs with different cesium doping concentration.

Figure 1a shows the schematic of the PSCs with a standard inverted structure of ITO/PEDOT:PSS/Perovskite/PCBM/BCP/Ag. The Pb(Ac)_2_-processed perovskite films (with and without cesium doping) were sandwiched between a poly(3,4-ethylenedioxythiophene) polystyrene sulfonate (PEDOT:PSS) hole transport layer and a 6,6-phenyl-C61-butyric acid methyl ester (PC_61_BM) electron transport layer. The J–V curves of the PSCs with 0–7.5 mol% cesium doping are exhibited in Figure 1b, with the photovoltaic parameters listed in Table 1. The reference PSCs without cesium doping had an average PCE of 14.1%, which is a standard value for Pb(Ac)_2_-processed inverted PSCs. When the perovskite was doped with 2.5 mol% cesium, the PCE increased to 15.04%. With 5 mol% cesium doping, the PCE further increased to 15.57%, which was mainly induced by the significant improvements in J_sc_ (from 20.16 to 21.08 mA cm^−2^) and FF (from 0.69 to 0.75). Doping with 7.5 mol% cesium degraded the PCE to 15.37%, indicating that 5% is the optimum cesium doping concentration for maximizing the PCE. As shown in Figure S1, we measured the J–V curves scanned in the reverse and forward directions at a scan rate of 200 mV s^−1^. The J–V curve of the reverse scan was almost the same as that of forward scan, indicating a negligible hysteresis of the device. To investigate the hysteresis deeply, dynamic J–V scans with calculation of the hysteresis index [17] are required, which is beyond the scope of this study.

**Figure 2:**
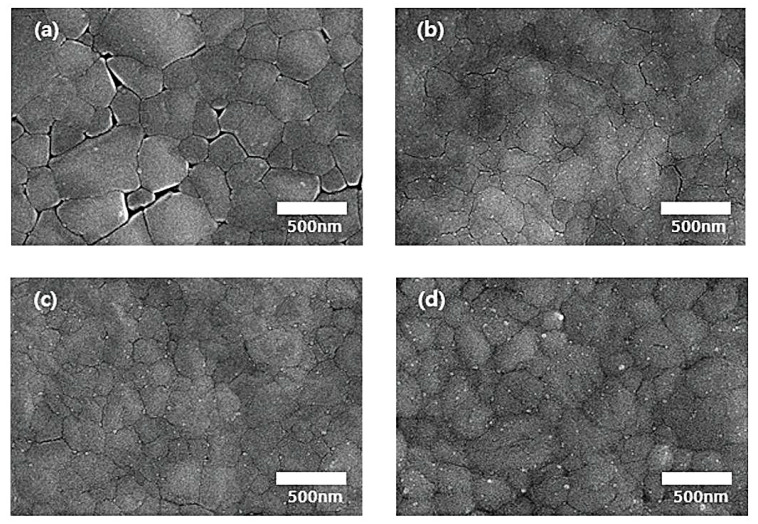
Top-view SEM images of perovskite films with (**a**) 0, (**b**) 2.5, (**c**) 5, and (**d**) 7.5 mol% cesium doping concentration.

To identify the origin of the improved PCE by cesium doping, we investigated the morphology of the 5%-Cs-doped and undoped perovskite films. As is evident in the surface SEM images in Figure 2a, the undoped perovskite film had poor surface coverage with many pinholes. A perovskite layer processed from a Pb(Ac)_2_-based precursor showed a similar surface morphology with some flaws, which might be caused by MA and halide deficiencies as shown in [13]. Figure 2a,d show that with increasing cesium doping concentration, the coverage of the perovskite layer onto PEDOT:PSS increased. As shown in Figure 2c, the 5 mol%-cesium-doped perovskite film had a dense and uniform morphology with full surface coverage. AFM images, shown in Figure S2, further confirm the increased surface uniformity with cesium doping; the root mean square roughness of the cesium-doped perovskite film is 8.6 nm, which is much lower than that of the pristine perovskite (14.6 nm). Furthermore, we found that the cesium-doped perovskite film showed less lateral grain boundaries compared to the pristine perovskite film. As discussed in previous studies, the pinholes in the perovskite film trap carriers, which further increase the charge recombination in the PSCs [18]. The SEM and AFM measurements indicate an improved morphology of the perovskite film upon cesium doping, which explains the PCE improvement, where improved perovskite seeding may be induced by the cesium addition [16]. These seeds might later turn into nucleation sites for further growth of perovskite during crystallization, which results in denser grains [16]. A similar process was found by Li et al. where MAI-modified PbS nanoparticles behaved as growth seeds for highly compact perovskite films [19]. To prove this mechanism, we characterized the crystallinity of the pristine and cesium-doped perovskite films.

**Figure 3:**
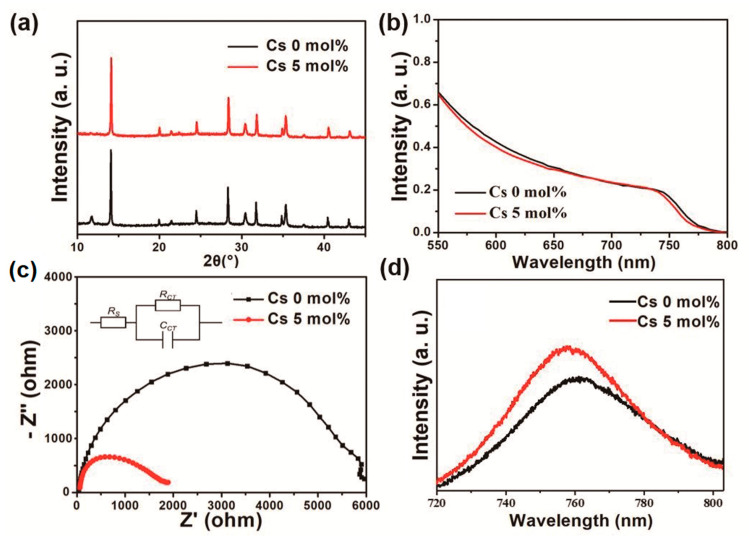
(**a**) XRD patterns. (**b**) Ultraviolet–visible absorption spectra of the perovskite films with different cesium doping concentrations. (**c**) Nyquist plots of the PSCs without and with 5% cesium doping with a bias of 0.8 V. (**d**) Photoluminescence (PL) spectra of the perovskite films without and with 5 mol% cesium doping.

Figure 3a compares the XRD spectra of the cesium-doped and pristine perovskite films. All peaks in the XRD patterns show the presence of the CH3NH3PbI3 tetragonal crystal structure [20]. It can be seen that the intensity of the (110) peak at 14° of the cesium-doped perovskite film is higher than that of the undoped one. Moreover, the peak at about 12°, which relates to the (001) lattice planes of hexagonal PbI_2_, is dramatically reduced with cesium doping. This indicates that the decomposition of the perovskite to PbI_2_ was suppressed by cesium doping [21,22]. In the UV–vis light absorption (Figure 3b), a small blue shift can be observed with cesium doping, indicating a slightly increased optical bandgap, in good agreement with previous studies [23].

**Figure 4:**
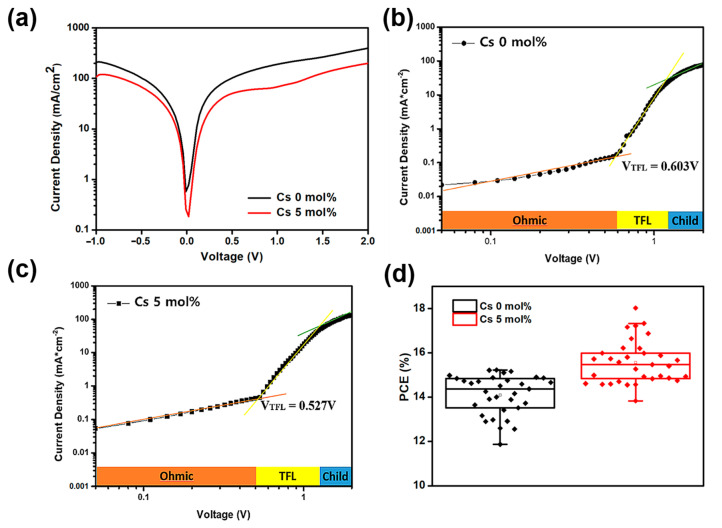
(**a**) Dark J−V characteristics of the PSCs with and without 5 mol% cesium doping. SCLC of the PSCs (**b**) without and (**c**) with cesium doping. (**d**) PCE distribution box chart of the PSCs without and with 5% cesium doping.

We also conducted EIS for the PSCs under one sun illumination to obtain the resistance information upon cesium doping. Figure 3c shows the Nyquist plots that are fitted with the equivalent circuit, which is shown in the inset. After fitting, the series resistance (R_s_), charge recombination resistance (R_ct_), and chemical capacitance (C_ct_) of the films could be obtained and the values of them are listed in Table S1. The R_s_ value for the case with 5 mol% Cs (60.5 Ω) is 33.0% lower than that without Cs doping (90.3 Ω), which contributes to the enhancement of J_sc_ and FF. The C_ct_ values, which are associated with the densities of space charges at the interfaces, are similar for the cases with and without 5% Cs doping (2.9 × 10^−9^ F and 3.0 × 10^−9^ F, respectively). The R_ct_ of 5% Cs-doped sample (3198 Ω) is lower than that of undoped sample (6653 Ω). Because the lower R_ct_ indicates the larger electron recombination at the interfaces, the R_ct_ values predict the higher leakage current and the lower J_sc_ for the Cs-doped samples. However, our experimental results show that the doping of 5% Cs reduces the leakage current, as explained below, and increases the J_sc_ (see Figure 4b). The experimental results of previous studies are also controversial; some studies reported that J_sc_ increases with R_ct_ [24,25,26], while other studies reported the increase in R_ct_ reduced J_sc_ [27,28,29]. We speculate that R_ct_ in our circuit model may not correctly represent the recombination resistance; R_ct_ in the circuit model was extracted from a high frequency impedance semicircle, whereas some previous studies insisted that R_ct_ is related to both high and low frequency semicircles [30,31,32]. Further studies using more sophisticated circuit models are required to obtain the more accurate R_ct_. Figure 3d shows the PL spectra for the 5 mol%-Cs-doped and undoped perovskite films on glass substrates. Evidently, the PL peak of the cesium-doped perovskite film was slightly blue-shifted to 756 nm (the PL peak of the pristine perovskite film is at 760 nm), which is consistent with the UV–vis absorption results in Figure 3d. The intensity of the PL peak of the cesium-doped perovskite film is 24% higher than that of the pristine perovskite film, which indicates decreased surface-trap states (related to non-radiative PL recombination) and increased perovskite crystallinity (consistent with the SEM results) [33].

Figure 4a showed the dark J–V characteristics of the 5 mol%-Cs-doped and undoped PSCs. The cesium-doped PSC shows smaller leakage current than the reference PSC without cesium doping across the voltage range 0 to −1.0 V. To analyze the trap density of the perovskite films with cesium doping, we measured the SCLC of the hole-only devices described above [34,35]. As shown in Figure 4b,c, the J−V curve can be divided into three regions.

The first segment at low bias (<0.4 V) is the ohmic region, in which the current density shows the almost linear increase with the voltage [36]. The second segment is called the trap-filled limit (TFL) region, in which the current density has rapid nonlinear growth, indicating the TFL in which the injected carriers deactivate available trap states [36]. At high voltages, the current density increases slowly, which is referred to as the Child’s regime. The TFL voltage (*V_TFL_*) is the voltage where the ohmic and TFL current curves intersect. The trap density ($n_{trap}$) can be calculated from V_TFL_ using the following equation [37];

(1) $$V_{TFL}=\;\frac{en_{trap}L^{2}}{2\epsilon_{0}\epsilon }$$

where *L* is the perovskite film thickness, *ϵ* (≈5.7565) is the relative dielectric constant of the CH_3_NH_3_PbI_3_ perovskite film [38], $\epsilon_{0\;}$ is the vacuum permittivity, and *e* is the elementary charge. As a result, the $n_{trap}$ values of the undoped and 5 mol%-Cs-doped devices are 5.8 × 10^16^ cm^−3^ and 3.6 × 10^16^ cm^−3^, respectively. The reduced trap density in the cesium-doped sample can be explained by the reduced pinholes and improved crystallinity of the perovskite layers (shown in Figure 2). In Figure 4d, it is shown that adding 5 mol% cesium enhanced the average PCE.

**Figure 5:**
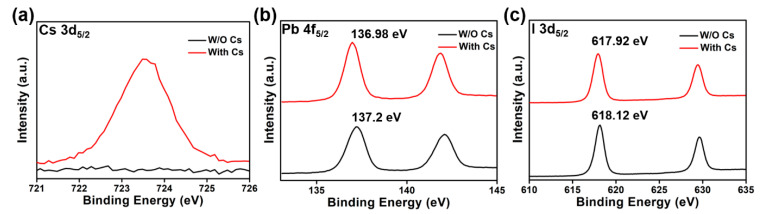
X-ray photoelectron spectroscopy (XPS) results for perovskite films without and with 5% cesium doping: (**a**) Cs 3d_5/2_, (**b**) Pb 4f_5/2_, and (**c**) I 3d_5/2_.

XPS spectra for the 5 mol% cesium-doped perovskite film (Figure 5a) show the Cs 3d_5/2_ peak at 724.41 eV, confirming the presence of cesium in the sample. In Figure 5b, the cesium doping slightly increases the binding energy of Pb 4f_5/2_ from 137.24 to 137.86 eV. For I, the 3d5/2 peak is also blue-shifted by the cesium doping from 618.16 to 618.8 eV, as shown in Figure 5c. We speculate that the doped cesium atoms cause local distortion in the lattice, which may affect the binding energies of the Pb and I ions.

Our results demonstrate that Cs doping is effective for improving the crystallinity and morphology of Pb(Ac)_2_-based perovskite layers, suppressing the formation of secondary phases such as PbI_2_. Thus, Cs doping is promising for enhancing the PCEs of Pb(Ac)_2_-based PSCs by improving the quality of perovskite films.

## 4. Summary

In this study, it is proved that suitable amounts of cesium can improve the film morphology and crystallinity of Pb(Ac)_2_-based perovskite films and adjust the electrical properties of the photoactive layer of perovskite for extracting more charge. PSCs based on these Pb(Ac)_2_-based perovskite films were demonstrated with a PCE, V_oc_, J_sc_, and FF of 15.57%, 0.98 V, 21.08 mA·cm^−2^, and 0.75, respectively. Additionally, the optimized devices showed negligible hysteresis in the forward and reverse J–V scans. This research shows that a perovskite precursor based on lead acetate is a promising source to achieve highly efficient PSCs, and further improvements will be possible through subtle tuning of the chemical composition.

## Figures and Tables

**Table 1:** Average photovoltaic parameters of the MAPbI_3_ PSCs based on perovskite precursors with 0 mol%, 2.5 mol%, 5.0 mol%, and 7.5 mol% cesium doping.

Cesium Doping Concentration (mol%)	V_oc_ (V)	J_sc_ (mA cm^−2^)	FF (%)	PCE (%)
0	0.97	20.16	69	14.10
2.5	0.98	21.84	71	15.04
5	0.98	21.08	75	15.57
7.5	1.01	20.8	75	15.37

## Data Availability

Data sharing is not applicable to this article.

## Supplementary Materials

The following are available online at https://www.mdpi.com/1996-1944/14/2/363/s1, Figure S1: Reverse scan and forward scan J–V curve of 5% Cs-doping device; Figure S2: Tapping-mode AFM height images of (a) the pristine and (b) Cs-doped perovskite films.; Table S1: Fitted values of the equivalent circuit parameters from dark Nyquist plots of devices without and with 5% Cs.
