# Supplementary material for: Cesium Doping for Performance Improvement of Lead(II)-acetate-Based Perovskite Solar Cells
Source: Materials (Basel). 2021 Jan 13;14(2):363. doi: 10.3390/ma14020363 (PMC7828501; doi:10.3390/ma14020363)
Supplement: Supplementary file 1 [file materials-14-00363-s001.pdf]

materials

MDPI

Supplementary Material

Cesium Doping for Performance Improvement of Lead(II)-Acetate-Based Perovskite Solar Cells

Min-Seok Han [1], Zhihai Liu [2], Xuewen Liu [1], Jinho Yoon [1] and Eun-Cheol Lee [1,3,*]

Department of Nano Science and Technology, Graduate School, Gachon University, Gyeonggi 13120, Korea; hanminsuk7@naver.com (M.-S.H.); arelaII5960@gmail.com (X.L.); wlsgh9838@naver.com (J.Y.)

[2] School of Opto-Electronic Information Science and Technology, Yantai University, Yantai 264005, China; zhliu@ytu.edu.cn

[3] Department of Physics, Gachon University, Gyeonggi 13120, Korea;

* Correspondence: eclee@gachon.ac.kr; Tel.: +82-31-750-8752; Fax: +82-31-750-8769

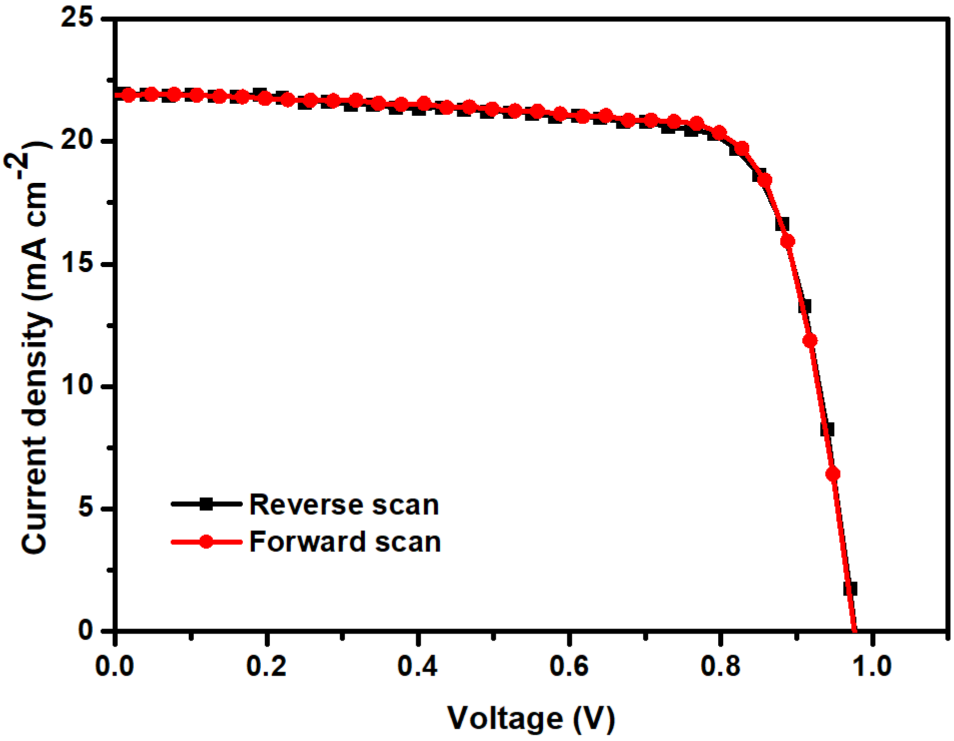

Figure S1. Reverse scan and forward scan *J-V* curve of 5% Cs-doping device.

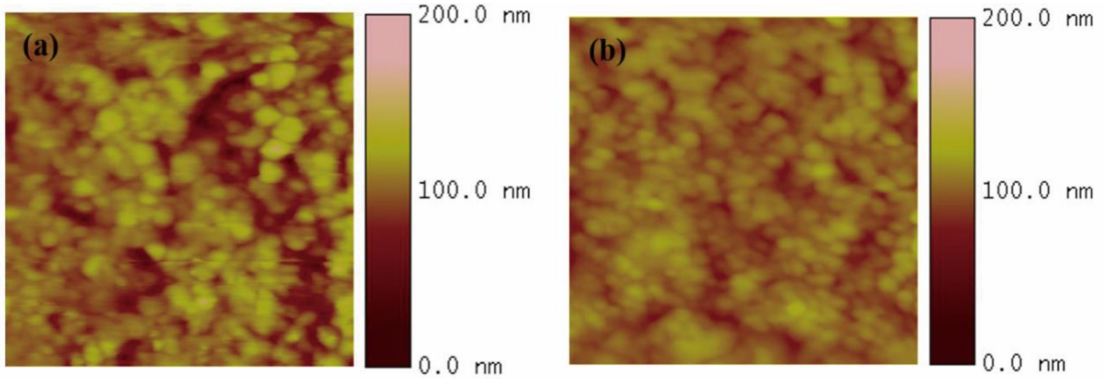

Figure S2. Tapping-mode AFM height images of (**a**) the pristine and (**b**) Cs-doped perovskite films.

Table S1. Fitted values of the equivalent circuit parameters from dark Nyquist plots of devices without and with 5% Cs.

**Cs (mol. %)**	**R_S (Ω)**	**R_{CT}（Ω）**	**C_{CT} （F）**
0%	90.3	6653	3.0×10^{-9}
5%	60.5	3198	2.9×10^{-9}
